# Rare Presentation of Sinonasal Synovial Sarcoma With Frontal Sinus and Limited Intracranial Extension Associated With Probable Paraneoplastic Vasculitis: A Case Report

**DOI:** 10.1155/cris/8837721

**Published:** 2026-05-29

**Authors:** Afnan W. M. Jobran, Ibrahim Alzatari, Yazan Abugharbieh, Mohammed Abdulrazzak, Hasan Arafat

**Affiliations:** ^1^ Faculty of Medicine, Al Quds University, Jerusalem, State of Palestine, alquds.edu; ^2^ Radiology Department, Al-Ahli Hospital, Hebron, State of Palestine, ahlihospital.com; ^3^ Faculty of Medicine, University of Aleppo, Aleppo, Syria, alepuniv.edu.sy; ^4^ Cancer Care Center, Augusta Victoria Hospital, Jerusalem, State of Palestine, kav-krankenhaus.de

**Keywords:** chemotherapy, diagnosis, MRI, synovial sarcoma, vasculitis

## Abstract

Synovial sarcoma is a high‐grade soft‐tissue sarcoma that commonly affects adolescents and young adults. Involvement of the head and neck region is rare, and tumors arising from the sinonasal tract are particularly uncommon and diagnostically challenging. We report the case of a 38‐year‐old male who presented with head trauma and decreased consciousness following a transient neurological episode while driving. Brain computed tomography revealed a lesion involving the left anterior ethmoidal air cells with associated old cerebral infarctions. Further magnetic resonance imaging demonstrated an aggressive enhancing sinonasal mass with limited intracranial extension, associated with radiologic findings suggestive of vasculitis and acute‐on‐chronic ischemic insults. Histopathological evaluation showed a spindle‐cell neoplasm considered most consistent with synovial sarcoma based on morphology and available immunohistochemical findings. The patient was started on neoadjuvant chemotherapy, after which the vasculitic findings showed regression, suggesting a probable paraneoplastic vasculitic association. Due to the complex anatomical location and local extension of the tumor, complete surgical resection was not initially feasible. Planned management included combined chemoradiotherapy with consideration of future surgical intervention. This case highlights the diagnostic and therapeutic challenges of sinonasal synovial sarcoma and emphasizes the importance of multidisciplinary evaluation, particularly in cases associated with unusual vasculitic and ischemic manifestations.

## 1. Introduction

Paraneoplastic vasculitides are rare syndromes associated with both hematological and solid malignancies, accounting for less than 5% of all vasculopathies. Their underlying pathogenesis remains poorly understood, and distinguishing true paraneoplastic vasculitis from coincidental associations remains challenging, because no specific biological markers have been identified [1].

A temporal relationship is often observed between the vasculitic process and the underlying malignancy, typically occurring within 12 months of cancer diagnosis. In many cases, the clinical course of the vasculitis parallels that of the malignancy, with improvement following successful cancer treatment and recurrence potentially indicating tumor progression [2].

Paraneoplastic vasculopathies are more commonly associated with hematological malignancies and only rarely occur in association with solid tumors, including sarcomas. Among solid malignancies, they are most frequently reported in lung, breast, colon, and renal carcinomas. Clinical manifestations usually involve small‐vessel vasculitis and may include rash, hematuria, myalgia, and arthralgia, with cutaneous vasculitis and polyarteritis nodosa representing the most commonly reported phenotypes [3, 4].

Herein, we report a rare case of sinonasal synovial sarcoma centered in the anterior ethmoidal air cells with frontal sinus, and limited intracranial extension, associated with probable paraneoplastic vasculitis complicated by ischemic cerebral infarctions. This case highlights the diagnostic and therapeutic challenges encountered in managing this unusual presentation. This report has been prepared in accordance with the SCARE 2023 criteria [5].

## 2. Case Presentation

**Figure 1:**
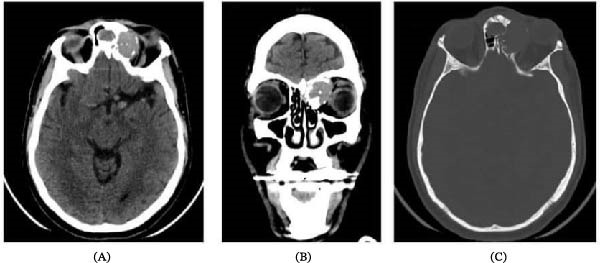
Nonenhanced brain CT images: (A) axial soft tissue window, (B) coronal soft tissue window, and (C) axial bone window; showing a well‐defined slightly hyperdense expansile lesion epicentered at the left anterior ethmoid air cells and left frontal sinus with extension into the medial extraconal space abutting the left medial rectus muscle. Several foci of calcification are seen within the lesion. The expansion of the lesion at the bone window (C) with marked thinning and destruction of the left medial orbital wall. Features are of aggressive lesions.

A 38‐year‐old male patient presented to the Accident and Emergency department after sustaining head trauma following a sudden episode of decreased level of consciousness while driving. Further history revealed recurrent attacks of headache, tongue heaviness, and deviation of the mouth during the month preceding presentation. Initial noncontrast brain computed tomography demonstrated a lesion involving the left anterior ethmoidal air cells, associated with irregularities of the middle cerebral arteries and old basal ganglia infarcts (Figure 1).

The patient was admitted for further evaluation. Laboratory investigations, including tumor markers and cytoplasmic antineutrophil cytoplasmic antibody (C‐ANCA), were unremarkable. Contrast‐enhanced computed tomography of the chest and abdomen showed no evidence of distant malignancy.

**Figure 2:**
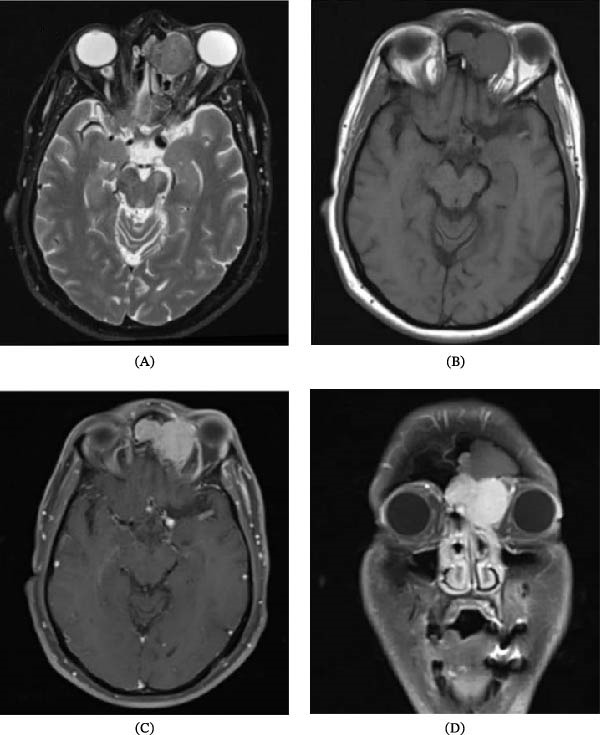
Multiplanar brain MRI with gadolinium images: (A) axial T2, (B) axial T2, (C) axial T1 with contrast, and (D) coronal T1 with contrast: the mentioned mass at the left anterior ethmoid air cells showing a solid signal intensity which is isointense to gray matter on both T1 and T2, with avid homogenous contrast enhancement.

**Figure 3:**
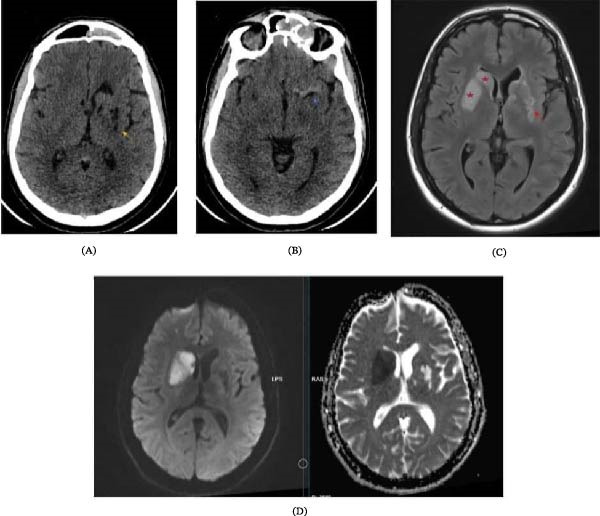
AXIAL CT and MRI images: (A and B) axial nonenhanced CT, (C) axial FLAIR MRI, and (D) DWI/ADC. Multiple hypodense areas are shown on CT (yellow arrow), representing areas of encephalomalacia with gliosis as shown on FLAIR images (red arrow). The hyperdense irregular left MCA (blue arrow). These areas are represent an old ischemic insult.

**Figure 4:**
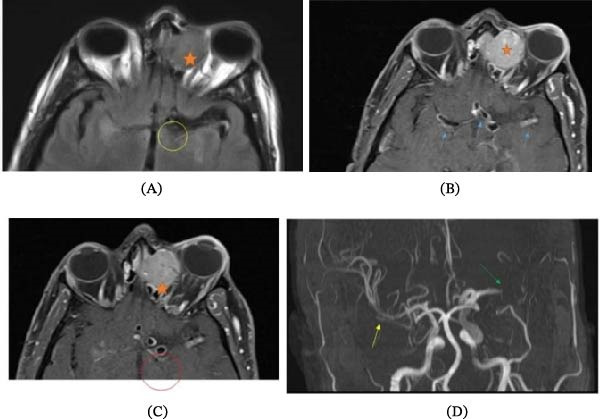
Focused MRI images: (A) focused axial FLAIR, (B and C) focused thin slices T1 with contrast, and (D) 3D TOF MRA. Concentric FLAIR hyperintense signal surrounding the left internal carotid artery (yellow circle). Concentric thickening and enhancement of the left internal carotid artery and both middle cerebral arteries (blue arrows). The left ethmoidal mass (orange stars). Correlation with MRA (D); there is mild ectasia of left ICA and M1 segment of left MCA, followed by severe narrowing of M2 segment, then by complete occlusion and paucity of the distal branches (green arrow). Irregularity and narrowing of the right MCA are also seen (yellow arrow).

Subsequent MRI with contrast demonstrated an aggressive, avidly enhancing lesion centered in the left anterior ethmoidal air cells with extension into the frontal sinus, orbit, and adjacent skull base structures (Figure 2) associated with signs of vasculitis and acute‐on‐chronic ischemic insults in the setting of an underlying vasculitic process (Figures 3 and 4).

A biopsy was subsequently performed using functional endoscopic sinus surgery (FESS) because of the highly vascular nature of the lesion. Histopathological findings were considered most consistent with synovial sarcoma based on spindle‐cell morphology and the available immunohistochemical profile. Detailed staining profiles and molecular translocation testing (SS18–SSX) were not available, as these investigations are not routinely accessible in our setting. The pathology report concluded that the findings were most consistent with synovial sarcoma. However, extended immunohistochemical characterization and molecular confirmation with SS18–SSX translocation testing were not available, representing an important diagnostic limitation.

The patient was started on neoadjuvant chemotherapy to downstage the locally aggressive tumor. Following treatment initiation, radiologic vasculitic findings demonstrated partial regression, suggesting a probable paraneoplastic vasculitic association related to the underlying malignancy. The patient is planned to undergo concomitant radiotherapy with the aim of further reducing tumor size and improving the feasibility of future surgical intervention.

## 3. Discussion

The synovial sarcoma typically develops in men in their second and third decades in the periarticular regions of the extremities. Sarcomas can occur as a result of Lynch syndrome, hereditary retinoblastoma, and neuroblastoma [6]. Of the 8%–10% of soft tissue sarcomas that it contributes to, 5%–10% affect the head and neck [7]. It has been identified in practically every part of the head and neck region; however, it is uncommon to find it in the paranasal sinus [6]. Additionally, only a few cases of ethmoidal sinus synovial sarcoma have been reported in the literature to date.

High‐grade sarcomas like synovial sarcoma are typically asymptomatic until compressive symptoms start to occur in surrounding areas, as in our case. In rare cases, diplopia or vision loss may also be present, along with nasal blockage, discomfort, earache, sore throat, and epistaxis [6].

Imaging studies may suggest malignant features; however, definitive diagnosis requires histopathological confirmation. In typical practice, confirmation of synovial sarcoma involves a combination of morphology, immunohistochemistry (such as TLE1, EMA, and cytokeratin), and, when available, genetic analysis for the SS18–SSX fusion gene. However, in many low‐resource settings, advanced molecular diagnostics are not routinely accessible. In such cases, diagnosis relies heavily on histomorphology and the immunostains available locally. Our case reflects this real‐world limitation, which is important to recognize when managing sarcomas in resource‐limited environments [8].

Resection with negative margins (R0), a fundamental tenet of oncological surgery, should be the goal, but it is challenging to accomplish in paranasal sinus surgery, particularly endoscopic sinus surgery, because of critical neurovascular structures adjacent to the tumor and microdebrider/piecemeal resection. Additionally, endoscopic surgery in these situations requires expertise; for these reasons, an open surgical approach is beneficial, especially in advanced cases. Intraoperative frozen‐section assessment may assist in evaluating surgical margins following tumor resection. Despite aggressive management, local recurrence rates remain high, particularly within the first 2 years following treatment [9]. In synovial sarcomas of the paranasal sinus and the skull base, the recurrence rate is noticeably quite high [10]. The grade, stage (bone invasion, size), and location all matter [11]. During the follow‐up period, routine endoscopic and radiographic examinations should be carried out. It is recommended to have a positron emission CT after 12 weeks of surgery to prevent false‐positive results.

Sarcomas benefitted from postoperative or final radiation [8]. With a median age of 64 years and a predominance of male patients, Andrä et al. [8] investigated 26 instances of head and neck sarcomas. 25 patients had high‐grade lesions, mostly angiosarcoma, malignant fibrous histiocytoma, and synovial sarcoma. With a median tumor size of 4.6 cm, the skull (including skin), paranasal sinus/orbit, and neck (including pharynx/larynx) were among the several anatomical components implicated. Only 38% of the 81% of cases that underwent surgical excision had tumor‐free margins; 23% had positive margins, and 19% had gross residual disease. All individuals received 66 Gy postoperative or final radiation, and sequential chemotherapy was given to half of the cases. They discovered 86% local control over a 5‐year period, while 82% of people survived [8]. Chemoradiation improves the prognosis for synovial sarcomas since they are chemosensitive, although overall survival is still dismal in high‐grade sarcomas, including synovial sarcoma [8].

In comparison, our patient was a 38‐year‐old male with a primary synovial sarcoma arising from the left anterior ethmoidal air cells, extending to the frontal sinus, with a measured tumor size of ~3.9 cm × 2.9 cm × 2.5 cm. The lesion demonstrated locally aggressive behavior with destruction of the left medial orbital wall, involvement of the cribriform plate, and minimal intracranial extension into the anterior cranial fossa, as well as extension into the postseptal extraconal orbital space, displacing the globe laterally. Histopathological evaluation confirmed a spindle cell mesenchymal neoplasm consistent with synovial sarcoma, with immunohistochemical positivity for CD99 and BCL2. Bone trabecular infiltration was also identified, supporting the aggressive nature of the tumor. Surgical resection with clear margins has not yet been achieved due to the complex anatomical location, and the patient is currently undergoing neoadjuvant chemotherapy with a plan for subsequent radiotherapy, likely within the standard range of 60–66 Gy. At the time of reporting, the patient remains under active treatment, and long‐term outcomes including survival and recurrence rates are not yet available.

## 4. Conclusion

Synovial sarcoma is a rare high‐grade soft‐tissue malignancy that uncommonly arises within the sinonasal tract. Because of its rarity and overlapping radiologic and histopathological features with other skull‐base tumors, accurate diagnosis remains challenging, particularly in resource‐limited settings where advanced molecular testing may not be available. This case highlights an unusual presentation of sinonasal synovial sarcoma associated with probable paraneoplastic vasculitis and ischemic cerebrovascular complications. Optimal management requires a multidisciplinary approach involving radiologic assessment, histopathological evaluation, systemic therapy, radiotherapy, and surgical planning when feasible.

**Nomenclature**
SS: Synovial sarcoma
CT: Computed tomography
MRI: Magnetic resonance imaging
FESS: Functional endoscopic sinus surgery.

## Funding

The authors declare no source of funding for this manuscript from any organization or any institution.

## Ethics Statement

The authors have nothing to report.

## Consent

Written informed consent was obtained from the patient and the patient’s family for publication of this case report and accompanying images. A copy of the written consent is available for review by the Editor‐in‐Chief of this journal upon request.

## Conflicts of Interest

The authors declare no conflicts of interest.

## Data Availability

The data that support the findings of this study are available from the corresponding author upon reasonable request.
